# Supplementary figures and images for: Concurrent HPV DNA testing and a visual inspection method for cervical precancer screening: A practical approach from Battor, Ghana
Source: PLOS Glob Public Health. 2023 Apr 20;3(4):e0001830. doi: 10.1371/journal.pgph.0001830 (PMC10118129; doi:10.1371/journal.pgph.0001830)

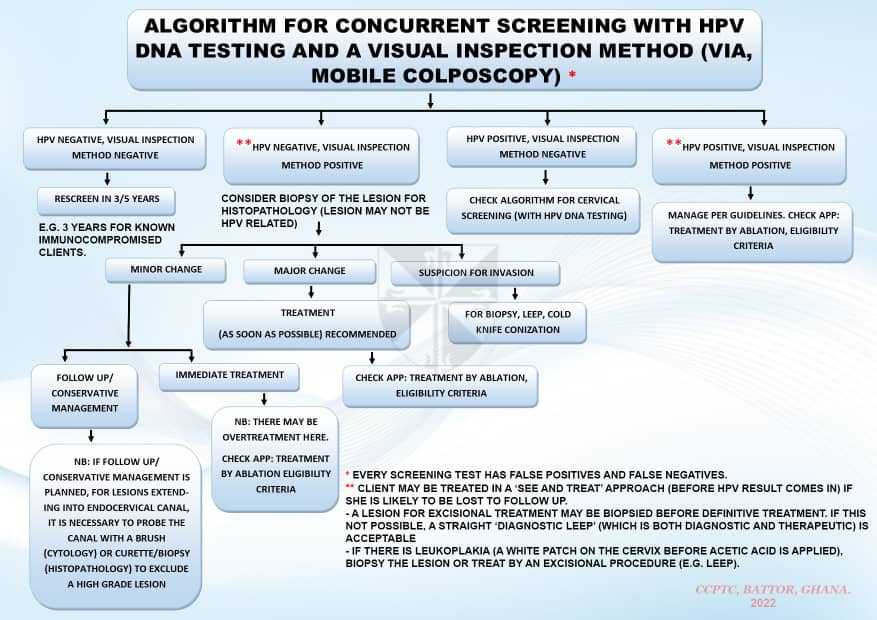

Supplement: S1 Fig — (TIF) [file pgph.0001830.s001.tif]

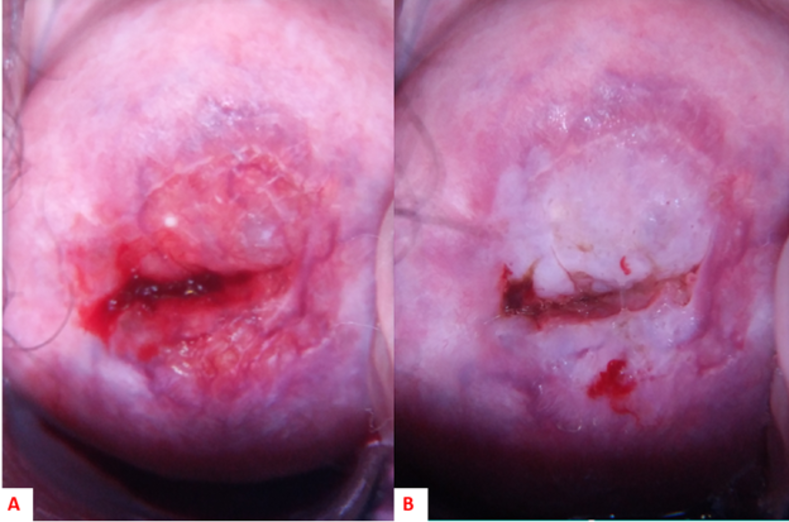

Supplement: S2 Fig — careHPV testing was performed concurrently with EVA colposcopy: (A) before applying acetic acid and (B) after applying acetic acid. careHPV–positive; EVA transformation zone type 3, dense aceto-whitening more anteriorly; treatment–LEEP; histopathology, CIN2. (TIF) [file pgph.0001830.s002.tif]

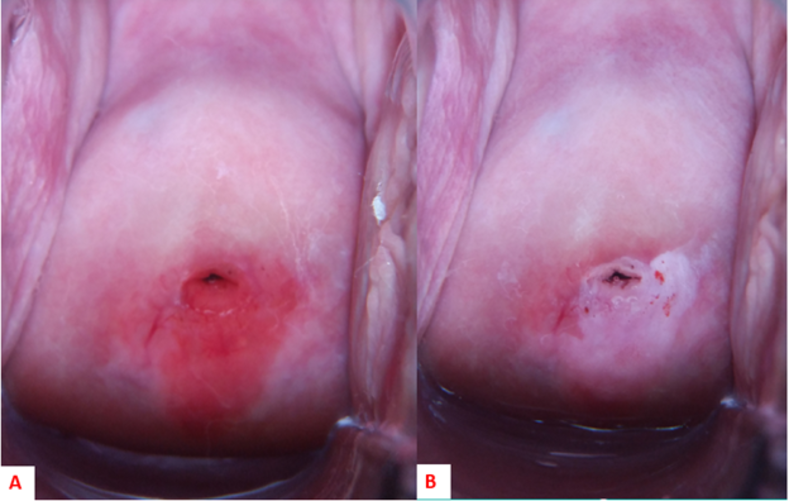

Supplement: S3 Fig — GeneXpert testing was performed concurrently with EVA colposcopy: (A) before applying acetic acid and (B) after applying acetic acid. GeneXpert–positive (others, P3); EVA transformation zone type 3, circumferential aceto-whitening, dense at the 1–3 o’clock position; treatment–LEEP; histopathology, CIN2. (TIF) [file pgph.0001830.s003.tif]

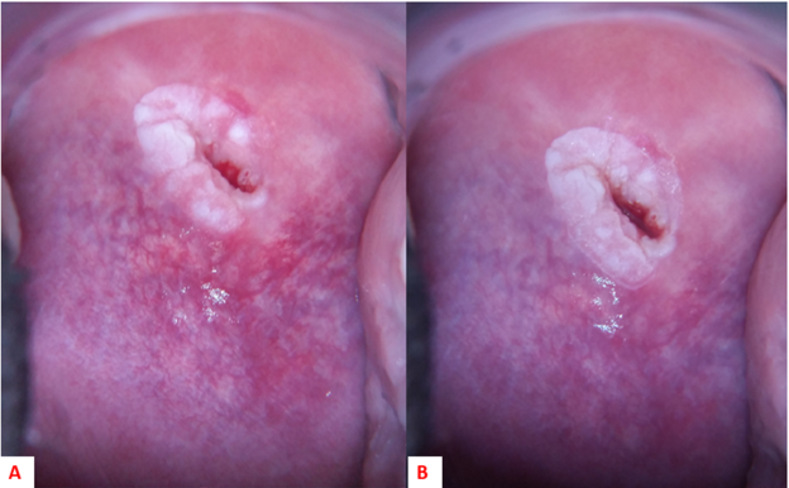

Supplement: S4 Fig — AmpFire hr-HPV testing was performed concurrently with EVA mobile colposcopy: (A) before applying acetic acid and (B) after applying acetic acid. AmpFire–positive for HPV 18; EVA–leukoplakia with circumferential dense aceto-whitening; treatment–LEEP; histopathology, CIN 3. (TIF) [file pgph.0001830.s004.tif]

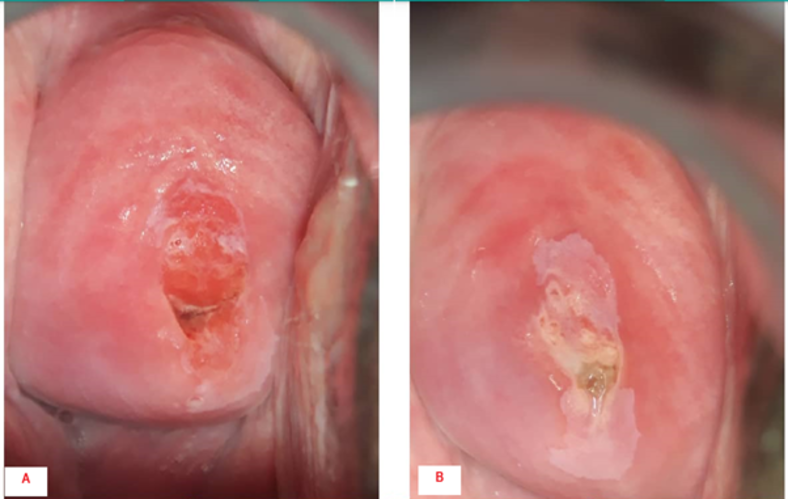

Supplement: S5 Fig — MA-6000 HPV DNA testing was performed concurrently with EVA colposcopy. MA-6000–positive for ‘other’ HPV type(s); EVA–adequate, transformation zone type 1, thin aceto-whitening on the anterior and posterior cervical lips; treatment–thermal coagulation. (TIF) [file pgph.0001830.s005.tif]
