# Supplementary material for: Concurrent HPV DNA testing and a visual inspection method for cervical precancer screening: A practical approach from Battor, Ghana
Source: PLOS Glob Public Health. 2023 Apr 20;3(4):e0001830. doi: 10.1371/journal.pgph.0001830 (PMC10118129; doi:10.1371/journal.pgph.0001830)
Supplement: Attachment — Submitted filename: Response to Reviewers.docx [file pgph.0001830.s006.docx]

| **Manuscript number: PGPH-D-22-02125**  **Journal: *PLOS Global Public Health***  **Title: Concurrent HPV DNA testing and a visual inspection method for cervical precancer screening: a practical approach from Battor, Ghana** | | |
| --- | --- | --- |
| **RESPONSES TO REVIEWER 1** | | |
| **Reviewer’s comment** | **Authors’ response** | **Line numbers** |
| 1. METHODS: a more precise description of when the visual inspection and the treatments were performed, as well as more information on the subgroup of women who underwent stand-alone HPV testing (i.e., all by self-sampling?), should be added. | We thank the reviewer for this comment. One hundred and thirty-five out of 1574 women in the standalone hr-HPV DNA testing group performed self-sampling. We have provided a brief description of self-sampling and sample storage in the methods subsection titled **“Cervical sample collection and HPV DNA screening”**. | 128–130  150–154 |
| 2. PARTICIPANTS: the characteristics reported in Table 1 on the number of children, the degree of education and the income earning identify these women as a selected group; this should be stated and the results commented also in relation to the generalizability of the findings. | We agree with the reviewer that the mentioned sociodemographic characteristics identify the participants of our study as a selected group. Thus, we have highlighted these recommendations by drawing a distinction between the group that underwent concurrent testing and recent estimates from the 2021 Ghana Population and Housing Census. We have also indicated the fact that our findings may not be generalizable to the entire general female population of Ghana under the **Strengths and limitations**. | 385–389 |
| 3. HIV INFECTION (page 8, line 190): the 54% HIV-negative fraction indicated does not take into account the fact that the HIV status was known for only slightly more than half of the included women, and should be better expressed as % of 2540 (that makes a 5% HIV positivity). Moreover, it should be interesting to separately report the outcomes in the 128 HIV-positive women. | Because the ‘unknown’ and ‘missing’ categories are indistinct, we have merged the frequencies for both categories under ‘Unknown’ in Table 1. In keeping with your recommendation, we have expressed the HIV positivity rate in the text as a proportion of 2538 women who knew their HIV statuses.  We have further included the positivity rates on EVA colposcopy (among 86 HIV positive women), VIA (among 41 HIV positive women), and hr-HPV DNA testing (among 127 HIV positive women) as nested proportions within Table 1 and highlighted that these rates tended to be higher for HIV positive women than for the entire study sample. | 220–221  227–229 |
| 4. DISCORDANT RESULTS: the HPV positivity rate was much higher than the positivity in visual inspection; the authors express concern for the risk of missing lesions in case of adoption of a stand-alone HPV testing; indeed, to this regard a very large amount of data in both high- and low-income countries exist demonstrating that HPV testing is more sensitive than cytology in detecting clinically relevant lesions (particularly CIN3+), although less specific; moreover, visual inspection has been shown to have a low specificity. Although a very small number of histological diagnoses have been performed in this study, the results (no dysplasia in 6/8 and CIN2 in 2/8 women HPV-negative & visual inspection positive) are in line with those data. The hrHPV-negative group, granted the use of clinically validated HPV assays, is not a clinically important group (as reported in page 17, line 353). | We agree partially that hr-HPV negative VIA positive group may not constitute a clinically important group if clinically validated HPV assays are used. However, we wish to create awareness among health workers in low-resource settings that HPV assays available (such as those used in our study) may be less sensitive, for instance, *care*HPV compared to hybrid capture 2, the gold standard. Thus, the increasing use of such platforms due to financial and resource limitations makes this group increasingly important. We have highlighted this issue among the limitations. | 380–385 |

| **RESPONSES TO REVIEWER 2** | | |
| --- | --- | --- |
| **Reviewer’s comment** | **Authors’ response** | **Line numbers** |
| 1. It is not clear from the data analyses and/or results how the four different high risk HPV tests compare to each other as this may have implications on the overall HPV prevalence reported in the study, especially if one test is more sensitive than the other - Additionally, it would have been interesting to see the high risk HPV positivity rate of all samples if they were all screened using a gold standard method in comparison to the 4 tests included in the study. | We appreciate the reviewer for these insightful comments. The four hr-HPV DNA tests differ from each other primarily in terms of target genotypes, collection medium, and the format of reading or reporting. To address these points, we have provided a separate section in the Methods section indicating these differences titled “**HPV DNA detection**”.  Further, we have disaggregated the hr-HPV detection rates according to platform in Table 1 and on Page 10, lines 229–232.  The reviewer also raises valid points that it would be interesting to compare our results for each test platform to those obtained via a gold standard. However, in our setting, gold standard methods such as the hybrid capture 2 platform are not currently available. It is for this reason that we resort to validated platforms such as *care*HPV and GeneXpert and have begun exploring AmpFire and MA-6000 as additional platforms due to resource limitations, throughput, ease of use, and sample batching within a context with a relatively small-scale cervical precancer screening service. Thus, at present, we are unable to provide such a comparison. | 161–172  229–232 |
| 2. This is a retrospective study that used a descriptive cross-sectional study design, however, the authors only used descriptive statistics to analyze their data. The reviewer suggests considering also using confidence intervals (CI) not just on odds ratio estimates, because point estimates alone do not tell a meaningful story, instead, include a 2-by-2 table for comparing the two screening approaches and perhaps also further include univariable and multivariable logistic regression models to adjust for any potential confounding variables. | While we appreciate the feedback and understand the importance of these techniques, we believe that incorporating these methodologies into our study falls outside the scope of our research objectives. We have at least three different potential lines of analysis: hr-HPV DNA positivity, EVA colposcopy ‘positivity’, and VIA ‘positivity’. As dependent variables, each of these would lead to their own set of independent factors and logistic regression models. We believe that by incorporating such analyses, we would lose focus of what we want to communicate with this paper. The objectives of our study were properly dealt with using the current descriptive approach and some other approaches suggested by the reviewers. The extra step of addressing potential risk factors will be focused on in a future article. | - |
| 3. Finally, great work on producing such a well-written and interesting paper. | We thank the reviewer for these kind words of encouragement. We also appreciate your continued contributions to scientific research. |  |

| **RESPONSES TO REVIEWER 3** | | |
| --- | --- | --- |
| **Reviewer’s comment** | **Authors’ response** | **Line numbers** |
| 1. Thank you for the submission of your work to the journal. Congratulations on the great work done in your country towards the elimination of cervical cancer. | Thank you for these kind words of encouragement. We also appreciate your continued contributions to scientific research. | - |
| 2. Since the authors used retrospective approach to the study there was a number of loss to follow up in the system, maybe if this was a prospective study design and patients knew that they were part of the study the loss to follow up could have better however this will mask this real life situation in the screening and treatment setting. In the results the authors mention risk factors however these were not mentioned in the methods and how they were collected and which of the data was retrieved from the system for analysis. The self sampling method used is not well explained in the methods, although self sampling method have been found to be as sensitive as the doctor collected samples, the self sampling devices differ and therefore this may bring bias in the results. This study outlines difficulties faced in low resource settings for cervical cancer screening and other screening programs. If women have to pay for their treatment the treatment is then bias and only those who can afford will receive treatment. | Thank you for reviewing our article and for making these keen observations about the potential limitations of our study. In the results, we described the proportions of women with two well-known risk factors for cervical cancer: smoking and HIV. Both of these were self-reported and not modeled via regression. In order not to confuse readers, we have modified the original sentence from:  “In terms of risk factors, most participants had never smoked (99.5%) and were HIV negative (54%).”  to  “As self-reported risk factors, most participants had never smoked (99.5%) and were HIV negative (54%). The HIV positivity rate among 2538 women with available information on HIV status was 5.0%.”  Further, 135 out of 1574 women in the standalone hr-HPV DNA testing group performed self-sampling. The same self-sampling (Evalyn self-sample brushes) were used by all 135 women, obviating any potential biases that could arise from using multiple self-sample devices. In keeping with your comment, we have provided a brief description of self-sampling and sample storage in the methods subsection titled **“Cervical sample collection and HPV DNA screening”**.  Regarding your comment about the difficulty faced by women in accessing cervical screening services in low-resource settings, we entirely agree that the cost of treatment remains a significant barrier for many women in our country, particularly due to the absence of a large-scale screening program. Thus, screening and treatment would be biased to only those who can afford it. Even among those who are able to avail such services, the absence of national guidelines and lack of financial coverage further bias treatment options. This was made clear within the limitations, in that many women tended to select thermal coagulation as a low-cost option to LEEP and histopathology, when given the choice. | 219–221  150–154 |
| 3. The issue of participants testing positive using VIA or EVA and testing negative with HPV could be due to the sensitivity of the test and the gene the test targets, its unfortunate that in the setting different HPV tests were employed and thus does not give a clear conclusion on HPV testing alone, furthermore this creates bias. | Regarding the reviewer’s comment on the bias arising from using different HPV tests, we acknowledge that this is a limitation of the study. Using different testing platforms could lead to differences in results and bias the results; however, in our setting, it was necessary to use different tests due to availability and accessibility issues, both of which are well addressed in the paper. For this reason, we have provided positivity rates for each testing platform in Table 1 and the results, and have highlighted this as a limitation. | 368–371 |
| 4. The VIA is subject to the user and therefore results may not be consistent throughout users. Then again, the VIA was conducted by the nurses while the colposcopy was conducted by the gynecologist (highly qualified and expert in the field). | We understand the concerns raised about the subjectivity of VIA. The nurses performed VIA and colposcopy in our setting. They are very well trained and experienced. VIA and colposcopy were supervised by specialist gynecologists, who served as a valuable and reliable reference points for the findings. The issue of subjectivity was also mentioned in the discussion in comparing the positivity rates for VIA and EVA. |  |
| 5. The limitations of the study are well outlined and explained by the authors however the conclusion is too long and the authors are discussing results instead of concluding based on the results. | We acknowledge that the conclusion was too lengthy and included some discussions and results. We have taken your feedback into consideration and revised it for conciseness with a focus on drawing conclusions based on our results. | 394–404 |
| 6. Lastly the S1 figure is already published elsewhere as it is. I have a concern though with publication of the images from colposcopy since this was a retrospective study the authors did not seek consent from the patients; however, I am not sure if they had consent from the hospital or clinic to publish the images. | The original S1 Figure has been deleted based on your recommendation. We have cited the article in which our research group first published the algorithm for cervical precancer screening with HPV DNA testing at the CCPTC.  Further, the Research Ethics Committee of the Catholic Hospital, Battor granted permission for the colposcopic images to be published on condition that they were anonymized. | 119–121 |

| **ADDITIONAL RESPONSES TO REVIEWER 3 FROM PDF ANNOTATIONS** | | |
| --- | --- | --- |
| **Reviewer’s comment** | **Authors’ response** | **Line numbers** |
| 1. **Line 35:** Please remove "or" and put "and" | We have corrected the original statement from:  The rates of EVA or VIA ‘positivity’ were 8.8% (95% CI, 7.0–11.0) and 2.0% (95% CI, 1.6–2.5), respectively, while the hr-HPV-positivity rate was 17.9% (95% CI, 16.7–19.0).  to  “The rates of EVA and VIA ‘positivity’ were 8.6% (95% CI, 6.7–10.6) and 2.1 (95% CI, 1.6–2.5), respectively, while the hr-HPV-positivity rate was 17.9% (95% CI, 16.7–19.0).” | 35–36 |
| 2. **Line 37:** Please rephrase this statement it does not make sense. e.g .....women in the cohort positive for hr HPV DNA and visual inspection concurrently. | In order not to confuse readers, we have refrained from using the word ‘concurrent’ to describe results or outcomes of testing. Instead, we have only used it in referring to ‘combined’ hr-HPV DNA testing and either form of visual inspection. Also, because women with negative findings on both methods were not clinically important, we present only the rate for that group and the rate and 95% CI for hr-HPV negative but visual inspection positive women. | 37–40 |
| 3. **Line 114:** However, the authors do not write this as the type of study | We have indicated that this study utilized a retrospective design in both the abstract and methods sections. | 33–35  131–134 |
| 4. **Line 125:** Please add “go” | We have corrected the original statement. | 124–126 |
| 5. **Line 130:** How many women were enrolled this information should be placed here. | We have revised the original statement. | 131–134 |
| 6. **Line 133:** The authors mentioned that the women paid for only EVA. | In order for nurses at our facility to gain more experience with VIA, we provided VIA at no additional cost to the women during the study period. They, however, paid for EVA colposcopy and hr-HPV DNA testing. This has been corrected within the text. | 124–126  362–363 |
| 7. **Line 134:** What kind of data was captured? | In the revised manuscript, we have included a separate subsection under the methodology titled “Variables and outcomes” describing what kind of data were captured and the outcomes of interest. | 138–144 |
| 8. **Line 138:** The authors mentioned that there were women who also had self-collected samples, how were these sample collected and with what and preserved in what? | One hundred and thirty-five out of 1574 women in the standalone hr-HPV DNA testing group performed self-sampling. We have provided a brief description of self-sampling and sample storage in the methods subsection titled **“Cervical sample collection and HPV DNA screening”**. | 150–154 |
| 9. **Line 143:** Do all the tests detect and genotype the same types of HPV? | The four hr-HPV DNA tests differ from each other primarily in terms of target genotypes, collection medium, and the format of reading or reporting. To address these points, we have provided a separate section in the Methods section indicating these differences. | 161–172 |
| 10. **Line 191:** The results suggests that not women underwent HPV testing and VIA at the same time. Only those that were HPV positive had undergone EVA colposcopy. Therefore, the word concurrent is misleading in this research. | In order not to confuse readers, we have refrained from using the word ‘concurrent’ to describe results or outcomes of testing. Instead, we have only used it in referring to ‘combined’ hr-HPV DNA testing and either form of visual inspection. | 224–227 |
| 11. **Table 1:** What is the difference between the unknown and missing status for HIV | Because the ‘unknown’ and ‘missing’ categories are indistinct, we have merged the frequencies for both categories under ‘Unknown’ in Table 1. In keeping with your recommendation, we have expressed the HIV positivity rate in the text as a proportion of 2538 women who knew their HIV statuses. | Table 1 |
| 12. **Line 233:** I suggest the authors rephrase the sentence and avoid using the words such as “concurrently negative” | In order not to confuse readers, we have refrained from using the word ‘concurrent’ to describe results or outcomes of testing. Instead, we have only used it in referring to ‘combined’ hr-HPV DNA testing and either form of visual inspection. | 270–273 |
| 13. **Line 343:** The conclusion is too long, there are discussions in here that should be in the discussion section | We acknowledge that the conclusion was too lengthy and included some discussions and results. We have taken your feedback into consideration and revised it for conciseness with a focus on drawing conclusions based on our results. | 394–404 |
